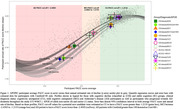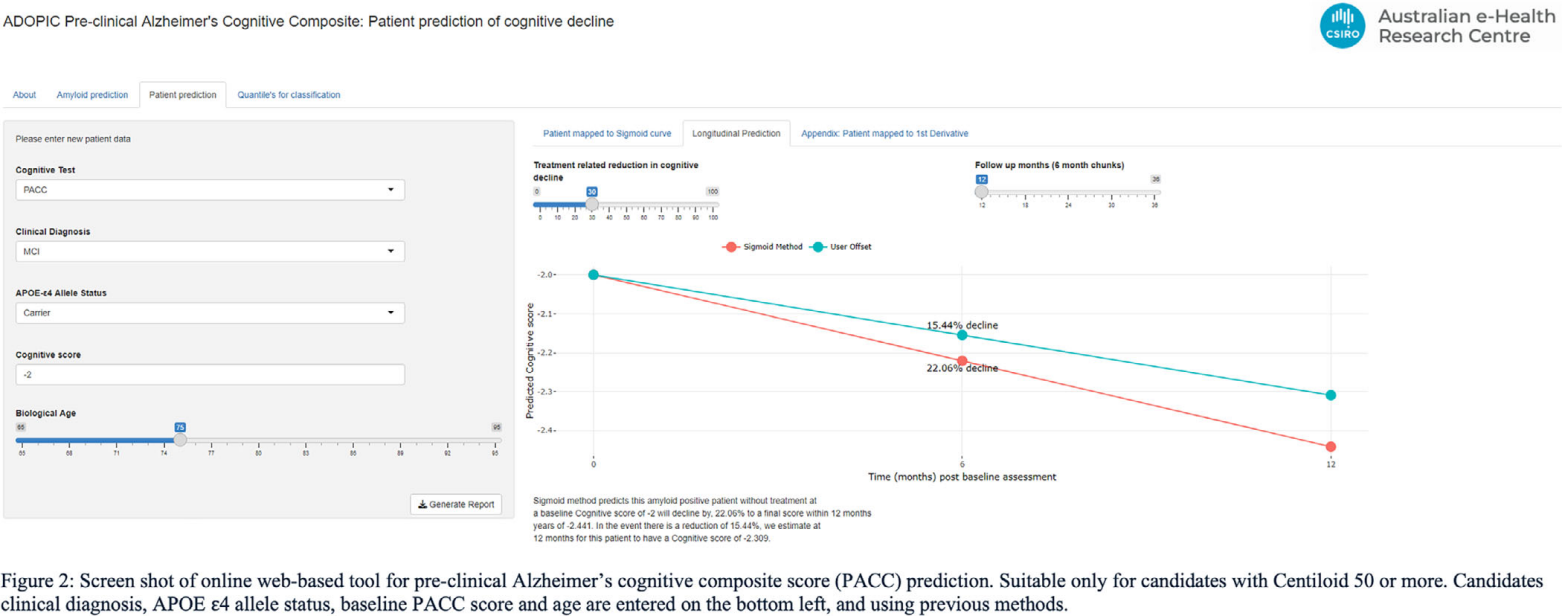# Early identification of participants at risk of cognitive decline: An online tool for patient prediction of cognitive decline

**DOI:** 10.1002/alz.091062

**Published:** 2025-01-03

**Authors:** Marcela Ines Cespedes, Rodrigo Canovas, Cai Gillis, Nancy N Maserejian, Paul Maruff, Christopher J Fowler, Stephanie R Rainey‐Smith, Ralph N Martins, Colin L Masters, James D. Doecke, The ADOPIC Consortium

**Affiliations:** ^1^ Australian E‐Health Research Centre, CSIRO, Herston, QLD Australia; ^2^ Australian E‐Health Research Centre, CSIRO, Melbourne, VIC Australia; ^3^ Biogen, Cambridge, MA USA; ^4^ Florey Institute of Neuroscience and Mental Health, Parkville, VIC Australia; ^5^ The Florey Institute of Neuroscience and Mental Health, The University of Melbourne, Parkville, VIC Australia; ^6^ School of Psychological Science, University of Western Australia, Crawley, Western Australia Australia; ^7^ Centre of Excellence for Alzheimer’s Disease Research and Care, School of Medical and Health Sciences, Edith Cowan University, Joondalup, Western Australia Australia; ^8^ Australian Alzheimer’s Research Foundation, Perth Australia; ^9^ Centre for Healthy Ageing, Murdoch University, Murdoch, Western Australia Australia; ^10^ Sir James McCusker Alzheimer’s Disease Research Unit (Hollywood Private Hospital), Perth, Western Australia Australia; ^11^ Macquarie University, Sydney, NSW Australia; ^12^ The Florey Institute of Neuroscience and Mental Health, University of Melbourne, Melbourne, VIC Australia; ^13^ School of Medical and Health Sciences, Edith Cowan University, Perth, Western Australia Australia; ^14^ The Australian e‐Health Research Centre, CSIRO, Brisbane, QLD Australia

## Abstract

**Background:**

The success of therapeutic options for treatment of Alzheimer’s disease (AD) and the growing emphasis for such treatment to commence in the pre‐clinical phase makes it necessary to have robust empirical models of clinical disease progression to understand findings from clinical trials, allow clinicians to evaluate effects of new drugs, and to select individuals for future trials. Such models have been developed from relatively small samples, with incomplete data/substantial loss to follow‐up. The ADOPIC consortium provides the largest complete AD natural history sample to date. We applied our sigmoid models of disease progression to cognitive data from ADOPIC, and developed an algorithm to predict cognitive change.

**Method:**

We developed an online application (Prediction of Alzheimer’s Disease Progression Tool, [PADPT]) to estimate decline in cognition over the course of AD within 2,861 ADOPIC participants (1,434 Cognitively unimpaired [CU], 342 MCI [Mild Cognitive Impairment], 211 AD, and 847 progressed from CU→MCI or MCI→AD), with ≥1 PET Amyloid (Aβ) scan and ≥36 months of cognitive assessment with the pre‐clinical Alzheimer’s cognitive composite (PACC). Individual participant slopes were defined according to age and gender using linear mixed effects models. Aβ+ (≥50CL) participant rates of decline vs their mean PACC score were investigated using quantile polynomial regression. Within PADPT, single visit information on age, clinical classification and *APOE* ε4 allele status is entered to compute the estimated annual rate of decline. Candidate results are mapped to the sigmoid curve to determine their predicted rate of cognitive decline over the chosen time period.

**Result:**

Quantile regression bands (QRB’s) aligned with participant cognitive decline. Individuals with stable cognition clustered to the top right of the plot (Figure 1). Mean slopes for *APOE* ε4 allele status separated QRB’s for CU and AD, but not for MCI groups. All participants allocated to cognitive decline groups (past the first sigmoid inflection point) demonstrated decline in PACC score that remained in the QRB’s, regardless of initial clinical classification.

**Conclusion:**

Used in conjunction with baseline profile information, the PADPT application can assist with clinical decision making both pre and post‐treatment and also identify suitable clinical trial candidates who, untreated, will show subtle decline in cognition.